# Individual structure mapping over six million trees for New York City USA

**DOI:** 10.1038/s41597-023-02000-w

**Published:** 2023-02-20

**Authors:** Qin Ma, Jian Lin, Yang Ju, Wenkai Li, Lu Liang, Qinghua Guo

**Affiliations:** 1 School of Geography, Nanjing Normal University, Nanjing, 210023 China; 2 Sierra Nevada Research Institute, University of California, Merced, CA 95348 USA; 3 School of Architecture and Urban Planning, Nanjing University, Nanjing, 210023 China; 4 School of Geography and Planning, Sun Yat-Sen University, Guangzhou, 510006 China; 5 Department of Geography and the Environment, University of North Texas, Denton, Texas 76203 USA; 6 Institute of Remote Sensing and Geographic Information System, Peking University, Beijing, 100871 China

**Keywords:** Environmental impact, Forest ecology, Urban ecology, Ecosystem services, Forestry

## Abstract

Individual tree structure mapping in cities is important for urban environmental studies. Despite mapping products for tree canopy cover and biomass are reported at multiple spatial scales using various approaches, spatially explicit mapping of individual trees and their three-dimensional structure is sparse. Here we produced an individual tree dataset including tree locations, height, crown area, crown volume, and biomass over the entire New York City, USA for 6,005,690 trees. Individual trees were detected and mapped from remotely sensed datasets along with their height and crown size information. Tree biomass in 296 field plots was measured and modelled using i-Tree Eco. Wall-to-wall tree biomass was mapped using relationships between field measurements and remotely sensed datasets and downscaled to individual trees. Validation using field-plot measurements indicated that our mapping products overestimated tree number, mean tree height and maximum tree height by 11.1%, 8.6%, and 5.3%, respectively. These overestimations were mainly due to the spatial and temporal mis-match between field measurements and remote sensing observations and uncertainties in tree segmentation algorithms. This dataset enables the evaluation of urban forest ecosystem services including regulating urban heat and promoting urban health, which can provide valuable insights for urban forest management and policy making.

## Background & Summary

Trees play an important role in urban ecosystems and provide a number of ecosystem services, including cooling effect through shading and evapotranspiration, absorbing and storing carbon dioxide and mitigating global warming, purifying air by trapping air pollutants, and reducing stormwater runoff by intercepting and absorbing rain^1–3^. Precise quantification of ecosystem services provided by urban trees is essential for urban planning and forest management. Most current studies mainly rely on optical imagery to quantify the spatial and temporal distribution of urban trees. Zhang, *et al*.^4^ identified tree canopy cover in 22 major cities in the US using aerial photos from National Agricultural Imagery Program (NAIP). O’Neil-Dunne, *et al*.^5^ generated a detailed Land Cover map over New York City (NYC) using NAIP imagery and Light Detection and Ranging (LiDAR) dataset. There are also studies using optical imagery-derived vegetation indices (such as Normalized Difference Vegetation Index, NDVI) to indicate greenness and vegetation density^6^. Both tree canopy cover and vegetation indices fail to quantify the three-dimensional (3D) structure of urban trees, such as tree height, crown size, volume, and biomass, without which precise planning and management of urban trees are difficult to implement.

Field survey and sampling is widely applied in urban environments to estimate tree structure and composition^7^. Many forest ecological models, such as i-Tree (https://www.itreetools.org/) and CUFR Tree Carbon Calculator, have been developed to assess ecosystem services and monetize ecosystem values provided by urban trees^8^. Nowak, *et al*.^9^ used the strategically sampled nearly 300 field plots in NYC and provided a systematic evaluation of ecosystem values provided by urban trees, including carbon storage, air pollution reduction, and reduced energy costs using the i-Tree model. This “field sample-model-estimation” method has been implemented in cities worldwide^10^, such as Syracuse United States^11^, Santiago Chile^12^, Bolzano Italy^13^, and Strasbourg France^14^, which provides comprehensive accounting of ecosystem services in citywide and neighbourhood scales. However, the accuracy of this approach varies across different case studies due to the uncertainties associated with field measures, sampling intensity and methods, and model parameters^15^. The lack of thorough individual tree information makes it difficult to derive practical tree management plans.

Individual tree survey from citizen science is becoming popular worldwide. For instance, in NYC, a citywide street tree census located and recorded a total of 692,923 street trees and their structural and health attributes, and this information is reported and updated on a public website (https://tree-map.nycgovparks.org/). Tree species at 203,024 locations across China have been collected and used to update vegetation mapping^16^. Citizen science-based tree data collection overcomes the limitation of labour and time costs in field surveys, but its quality is difficult to control when compared with data collected by professional forestry crews^17^. Moreover, citizen science-based surveys often cluster in conveniently accessible areas, such as road crossings or residential areas. However, a large portion of urban trees (e.g. approximately 90% of trees in NYC) grow in relatively remote or less accessible areas, where citizen’s accessibility is limited and phone-based GPS signal is not precise enough to locate individual trees. These datasets from citizen science can help with urban tree management to some extent, but extra efforts are needed to extend the sampling range and improve data quality.

LiDAR is an active remote sensing technology that can provide precise 3D quantification of tree structures^18^. Unlike optical imagery-based urban tree mapping that is often influenced by cloud cover^19^, shadows^20^, and saturation effect^21^, the LiDAR dataset can penetrate through vegetation surface and precisely delineate individual tree structures^22^. Many studies have successfully quantified tree height, crown size, and crown volume for individual trees from LiDAR data^23–26^. Individual tree segmentation from LiDAR data is usually the first step for tree structure estimation. A number of tree segmentation methods have been developed, including point-cloud based classification using top-down^27^ and bottom-up^28^ strategies; and Canopy Height Model (CHM) based tree segmentations, such as marker-controlled watershed segmentation^29^. The CHM is a rasterized LiDAR data that can efficiently characterize the canopy surface height by normalizing LiDAR points over the ground surface. Compared to point-cloud based tree segmentation methods that account for individual points, CHM-based method is more efficient in delineating over-story trees, although some LiDAR information from mid- or under-story is lost after rasterization^30,31^. Overall, CHM-based method is one of the most efficient methods for urban tree identification over large areas from airborne LiDAR datasets, as multi-story forests are less common in urban environments. Tree structural parameters can then be derived from LiDAR-derived metrics either directly or indirectly. Tree height, crown size, and volume can be estimated for each tree segment from either LiDAR CHM values or LiDAR point cloud^32,33^. Tree biomass is often indirectly estimated from LiDAR-based metrics and field measurements using the relationships between them^26,34,35^.

To overcome the insufficiency in urban tree structural mapping, we identified individual tree locations, and calculated tree height, crown size, crown volume, and biomass information from remotely sensed datasets and field measurements for the entire NYC. This study mapped over 6 million trees across five boroughs in NYC and provided spatially explicit information regarding tree density, individual tree size, and biomass. The potential applications of this dataset include but are not limited to (1) analysing spatial patterns of urban trees over different land cover and land use types and across census blocks, and to identify the inequity in urban green accessibility; (2) modelling the urban cooling effects under diverse 3D arrangements of trees and built-ups in real urban environments; (3) identifying areas that have least urban greens but most severe urban heat and pollution exposure; (4) prioritizing urban tree plantation and management; and (5) evaluating the ecosystem benefits and projecting their potential ecosystem values over time under different urban tree plantation and management strategies.

## Methods

### Study area and field data

**Fig. 1:**
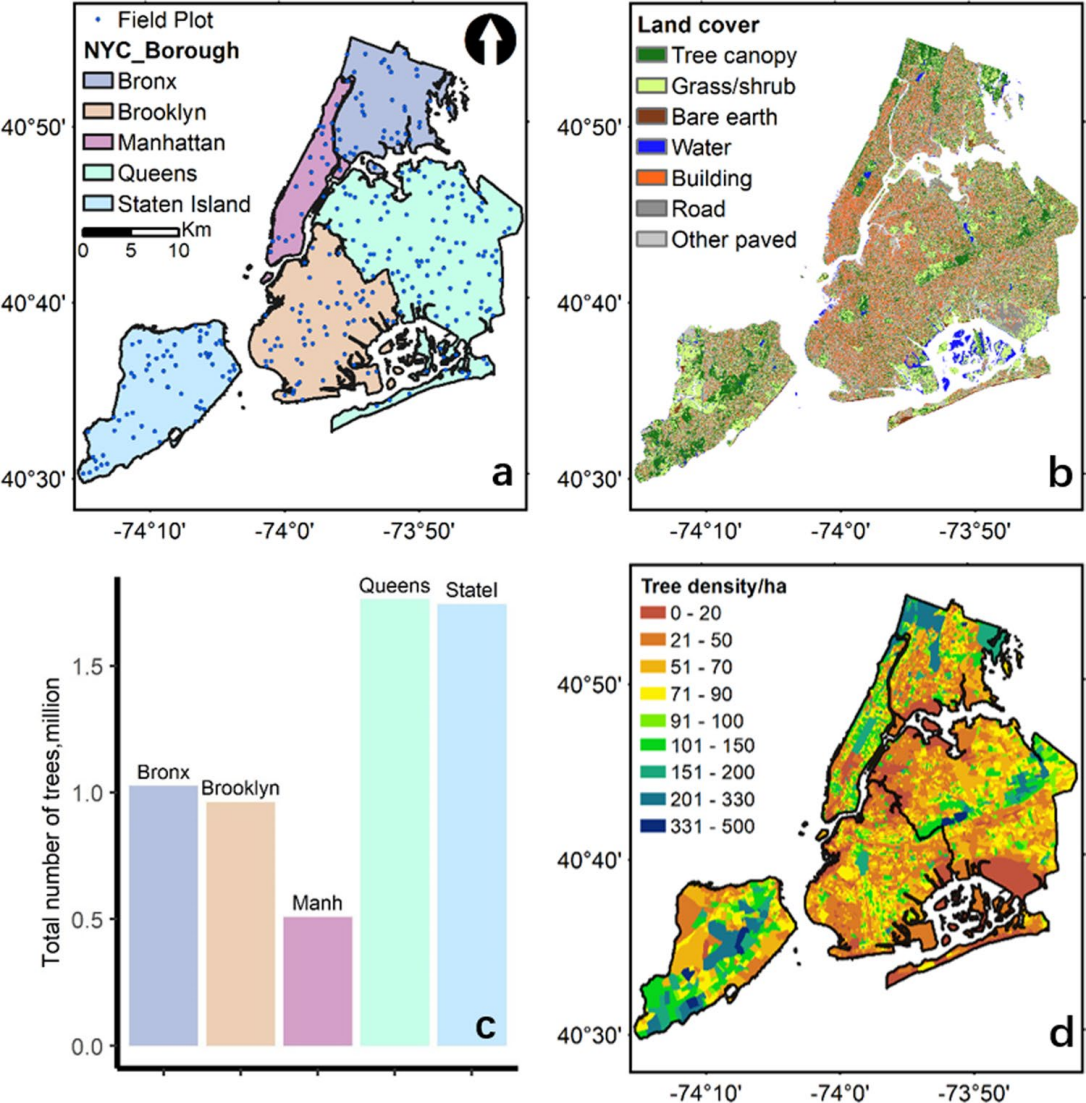
The distribution of field plots across five regions in New York City (NYC) borough (**a**). The land cover map over the entire NYC with seven land cover types (**b**). The summary of identified trees from remotely sensed datasets across five regions (**c**), and the tree density map over each block group in NYC (**d**).

The dataset was generated over NYC, located in the north-eastern United States (40.713° N, 74.006° W). NYC has a total area of 778.2 km^2^, which is composed of five boroughs, i.e. Brooklyn, Queens, Manhattan, Bronx, and Staten Island (Fig. 1). There were 296 field plots randomly sampled (Fig. 1a) and measured in the summer of 2013 over NYC following the i-Tree Eco protocols^9^ developed by the United State Department of Agriculture Forest Service (USFS). Each plot occupied a circular area of 404.7 m^2^. All the trees with a Diameter at Breast Height (DBH) larger than 2.54 cm were surveyed to record their tree height, species, DBH, and other structural attributes. Within all the 296 plots across NYC, there were 1,075 trees in 139 species surveyed. The species types with the top ten largest sample size were *Acer platanoides* (65 samples), *Cedrus species* (59 samples), *Ailanthus altissima* (58 samples), *Sassafras albidum* (56 samples), *Quercus alba* (51 samples), *Betula lenta* (42 samples), *Robinia pseudoacacia* (39 samples), *Acer rubrum* (38 samples), and *Hardwood species* (37 samples). Because the exact coordinates of individual trees were not collected, we mainly used the plot-level tree attributes (i.e. tree number, mean tree height, and maximum tree height) to validate LiDAR-derived products. Due to confidential requirements, the exact coordinates of field plots were not allowed to be released.

### Aerial image and land cover maps

A fine-resolution land cover dataset (0.91 m spatial resolution) provided via NYC OpenData (https://opendata.cityofnewyork.us/) was used to mask out non-vegetation areas. This land cover dataset was generated using an object-based image classification method^5^ from LiDAR data collected in 2010 and NAIP aerial imageries in 2009. This final land cover map includes seven classes, i.e., tree canopy, grass/shrub, bare earth, water, buildings, roads, and other paved surfaces (Fig. 1b). We regrouped the land cover map into vegetation (tree canopy and grass/shrub) and non-vegetation groups, and resampled the map into 1 m resolution to match with NAIP and LiDAR datasets. We also collected NAIP imagery in the summer of 2013 for tree structural estimation from the Google Earth Engine platform^36^. The NAIP image had a resolution of 1 m with four spectral bands (Red, Green, Blue, and Near Infrared). We further calculated the NDVI from the Red and Near Infrared bands of NAIP images for tree structural estimation.

### LiDAR data and processing

The LiDAR data were collected using a Leica ALS70 LiDAR system from two flight missions (https://noaa-nos-coastal-lidar-pds.s3.amazonaws.com/laz/geoid18/4920/index.html). The first LiDAR flight was taken on August 5^th^, 2013 at 2,286 m above ground level with an average side lap of 30%. The LiDAR data from this flight had a nominal pulse spacing of 0.91 m. The second flight was taken between March and April, 2014 at 2,286 m above ground level with an averaged side lap of 25% and a nominal pulse spacing of 0.7 m. According to the ground control survey, the LiDAR scan had a root mean square error accuracy of 9.25 cm. With up to 7 returns per pulse, the final LiDAR dataset has a point density of 5.9 points/m^2^.

The tree structural information was mainly generated from LiDAR-derived CHM. The CHM was the difference between Digital Surface Model (DSM) and Digital Terrain Model (DTM) generated from LiDAR point clouds using the Kriging interpolation method^37^. All the raster layers (CHM, DSM, DTM) were generated at 1 m resolution using the LiDAR360 software (GreenValley International). We generated a Tree Canopy Cover (TCC) map by masking out non-vegetation land cover types from areas with CHM values larger than 2 m. The TCC was a binary map with the value of one indicating tree cover and zero indicating non-tree cover at 1 m resolution. The non-vegetation areas were derived from the land cover map (Fig. 1b). The 2 m tree canopy height threshold was chosen by referencing a commonly accepted canopy height threshold^38^.

### Individual tree segmentation and feature estimation

Individual tree crowns were segmented from LiDAR-derived CHM using the Marker-controlled Watershed Segmentation algorithm. This algorithm was widely adopted for LiDAR-based tree crown segmentation^25,26,29^ because it takes the advantages of both region-growing and edge-detection methods^39^. Due to the relatively low LiDAR point density, the CHM contained abnormal pits even after masking out non-tree-canopy pixels. We applied a Gaussian filter with two standard deviations to smooth the CHM and fill these pits in CHM. Then the segmentation was applied with a 3 × 3 moving window. Both smoothing and segmentation were conducted using the System for Automated Geoscientific Analyses software^40^. To refine the segmentation results, we deleted small segments with an area smaller than 1 m^2^ (one CHM pixel), which was most likely to be noise in CHM. We also visually examined and manually re-segmented extremely large segments by assuming most tree crowns should not exceed an area of 200 m^2^. The final tree crown dataset only contains segments with a maximum CHM value no less than 5 m because vegetation with lower height was mostly likely to be non-tree. All the post-segmentation operations were conducted in ESRI ArcMap 10.8.

We estimated five tree structural features for each individual trees, which include tree top height, tree mean height, crown area, tree volume, and carbon storage. Tree top height (m) characterizes the height from ground to tree top, estimated as the maximum CHM value within each tree crown segment. Tree mean height (m) indicates the average height of the tree crown surface, calculated as the mean CHM values within each tree segment. Tree crown area (m^2^) is the total area of each tree crown segment. Tree volume (m^3^) is the volume of 3D space occupied by the tree crown^25^, which was calculated as the volume difference between crown surface (defined by CHM) and crown base (Eq. 1). Because the tree crown base height was difficult to estimate for individual trees due to the relatively low LiDAR point density, we used the 2 m to approximate the averaged crown base height according to Ma *et al*.^25^. The sensitivities of crown volume to the selection of crown base height from 1 m to 5 m was presented in the Technical Validation section.

1 $$Volume={\sum }_{i=1}^{n}\left(CHMi-crown\;base\;height\right)\times 1{m}^{2}$$

Where *CHMi* is the *CHM* values of the *i*_th_ pixels within a tree segment, *n* is the total number of pixels within a tree segment. 1*m*^2^ *is the area of each CHM pixel*.

The carbon storage (ton) was defined as the total carbon stock in both above- and below-ground biomass of each tree. The carbon storage was estimated in two steps: (1) calculating tree biomass from field measurements using allometric equations^41^; (2) running a regression between field measured carbon storage and LiDAR-derived tree structural features^42^ and applying the regression model to individual trees. In step (1), we applied species-specific allometric equations from i-Tree Eco database. There are more than 50 species-specific equations in i-Tree Eco, which can be summarised into four main equation forms with different coefficient values (Eqs. 2–5).

2 $$Biomass=\exp \left({\beta }_{1}+{\beta }_{2}\ast LN\left(DBH\right)+\frac{{\sigma }^{2}}{2}\right)$$

3 $$Biomass=\exp \left({\beta }_{1}+{\beta }_{2}\ast LN\left({{\rm{DBH}}}^{2}\ast {\rm{H}}\right)+\frac{{\sigma }^{2}}{2}\right)$$

4 $$Biomass={\beta }_{1}\ast \left(DB{H}^{{\beta }_{2}}\right)$$

5 $$Biomass={\beta }_{1}\ast \left({\left({{\rm{DBH}}}^{2}\ast {\rm{H}}\right)}^{{\beta }_{2}}\right)$$

Where *β*_1_ and *β*_2_ are species-specific coefficients, *DBH* is diameter at breast height, H is tree top height, *σ*^2^ is the variance of model errors, which is applied to correct the potential underestimations when back-transforming predictions from logarithmic scale to original scale. For other species that were not included in the i-Tree Eco database, the averaged results from the four equations were applied. These allometric equations (Eqs. 2–5) estimate the entire tree biomass including both above- and below-ground biomass, and the final carbon storage for each field plot was converted to carbon by a factor of 0.5^41^.

In step 2), we compared different regression models to simulate carbon storage at plot scale using LiDAR data and NAIP imagery. First, we compared the single variable regression for carbon storage from NAIP-derived NDVI, LiDAR-derived TCC, LiDAR-derived CHMmean and CHMmax, respectively. The four metrics were calculated at 1 m resolution, masked out non-vegetation areas, and aggregated over each field plot. TCC was calculated as the percentage area with tree cover (CHM >2 m). CHMmean and CHMmax were calculated as the mean and maximum of all CHM values within each plot. We compared different regression algorithms, including linear, exponential, and quadratic regressions. We also compared the modelling efficiency and accuracy between using single and multiple variables by combing all the attributes together using the Random Forest regression model. Using the optimal regression model, we generated a carbon density map at 20 m spatial resolution (each pixel size is similar to the plot size of 404.7 m^2^) by dividing the total carbon storage by the pixel size (400 m^2^) in the unit of ton/ha (Eq. 6). More details of carbon density estimation can be found in our previous publication^42^. The carbon storage for each tree was calculated as the product of crown area and crown density (Eq. 7).

6 $$Carbon\;density\left(ton/ha\right)=0.5\ast Biomass(ton)/\left(400\left({m}^{2}\right)\ast 0.0001\left(ha/{m}^{2}\right)\right)$$

7 $$Carbon\;storage\left(ton\right)=Crown\;area\left(ha\right)\ast Carbon\;density(ton/ha)$$

Where Biomass is the total biomass for each pixel, which was 400 m^2^. ha is short for hectare, which is 10000 m^2^.

We further quantified the uncertainty range in carbon storage estimation by propagating the potential error in carbon density regression to tree level carbon storage estimation. We first calculated the 95% confidence interval of the best carbon density regression model, and applied the confidence interval to carbon storage estimation for individual trees. The predicted the upper and lower values for individual tree carbon storage were given in the final dataset and summarized in Table 1.

**Table 1:** A summary of the individual tree carbon storage prediction (Carbon) and their lower (Carbon_lower) and upper (Carbon_upper) values. The minimum (min), maximum (max), mean, standard deviation (std), first quartile (q25), median, and third quartile (q75) values of individual tree carbon storage are presented.

*Tree carbon stock, ton*							
	*min*	*max*	*mean*	*std*	*q25*	*median*	*q75*
Carbon	0	5.771	0.172	0.238	0.035	0.086	0.210
Carbon_lower	0	5.436	0.162	0.224	0.033	0.081	0.198
Carbon_upper	0	6.107	0.182	0.252	0.037	0.091	0.222

### Block group level tree structure distribution mapping

Three sets of tree structural parameters were mapped at block group level, including tree density (the number of trees in each hectare), tree height (m), and carbon density (ton/ha). The mean values of tree density, tree top height, and carbon density within each block group of NYC. The block group boundary was downloaded from https://www.census.gov/geographies/mapping-files/time-series/geo/tiger-line-file.2014.html, which includes a total of 6392 block groups.

We also estimated the potential tree height and carbon density at the census block group level. We assumed the 95% of the tree height and carbon storage values within each block group at mapping time were their potential values, which most trees can achieve during their life time. Then, we calculated the difference in tree height and carbon density between potential values (95%) and mapping time values (mean) over each block group, and used them as the extra carbon storage that trees could achieve during their life time. It is to be noticed that in this study we did not consider the carbon loss by tree degradation or removal, or extra carbon gain through the tree planting and management.

## Data Records

### Tree structural maps and datasets

This study generated a Canopy Height Model (CHM) map over tree canopy covered area at 1 m spatial resolution for the entire NYC^43^ (10.6084/m9.figshare.20522895.v2). The CHM dataset was recorded in the unit of decimetre (dm) considering the precision of the LiDAR dataset (approximately 9 cm) and was saved in integer data format. Pixels with CHM values lower than 2 m were considered as non-tree, and masked out along with other non-vegetation land cover types. Pixels with CHM larger than 60 m were also masked out as non-trees to avoid misclassification with buildings since trees larger than 60 m were unlikely to exist in NYC. Therefore, the final CHM map has values ranging from 20 to 600 dm. We also provided a carbon density map over the tree canopy covered area for the entire NYC at 1 m spatial resolution in the unit of ton/ha. The Carbon density ranged from 0 to 410.1 ton/ha in float data format. Both CHM and Carbon Density maps are in GeoTiff format under the NAD83 (National Spatial Reference System 2011) Universal Transverse Mercator Zone 18 North projection.

The individual tree dataset includes tree location and tree crown in Shapefile formats under the same projection^43^ (10.6084/m9.figshare.20522895.v2). The tree crown dataset records the refined polygons segmented from LiDAR-based CHM. The attributes table of the tree crown polygons include the tree ID, polygon area (m^2^), tree top height (m), tree mean height (m), tree volume (m^3^), and carbon storage (predicted value, lower value, and upper value in ton). The tree location dataset records the center point of each tree crown with the tree ID information that can be linked to other attributes in the tree crown dataset. For easy display and usage, we divided the individual tree dataset into five regions, i.e. Brooklyn, Queens, Manhattan, Bronx, and Staten Island.

### Spatial distribution of tree height, tree density, and carbon storage

There were 6,005,690 trees identified from the remotely-sensed dataset over the entire NYC (Fig. 1). The Queens and State Island regions had over 1.7 million trees, which were 1.5 to 3.5 times of other regions. Highly dense trees (>200 /ha) clustered in the northeast of Bronx, middle of Queens, and middle-south of State Island, where most urban parks and natural areas existed (Fig. 1d).

**Fig. 2:**
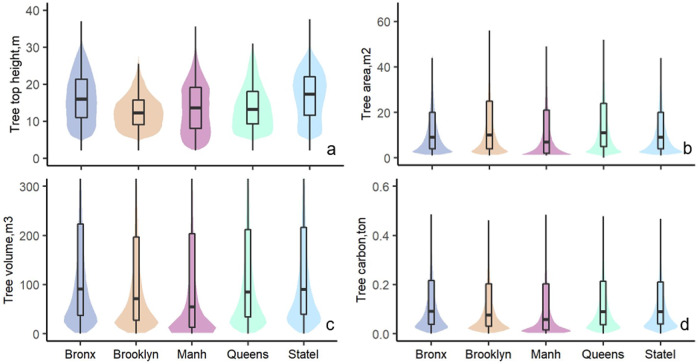
The violin and boxplot of tree top height (**a**), tree crown area (**b**), tree volume (**c**), and carbon (**d**) across each region in New York City. The upper and bottom whiskers of the boxplot indicate the minimum (fist quartile-1.5interqurtile range) and maximum (third quartile + 1.5interquartile range), and the box values indicate the first quartile, median, and third quartile values with each region. Outliners were not presented in this plot.

**Fig. 3:**
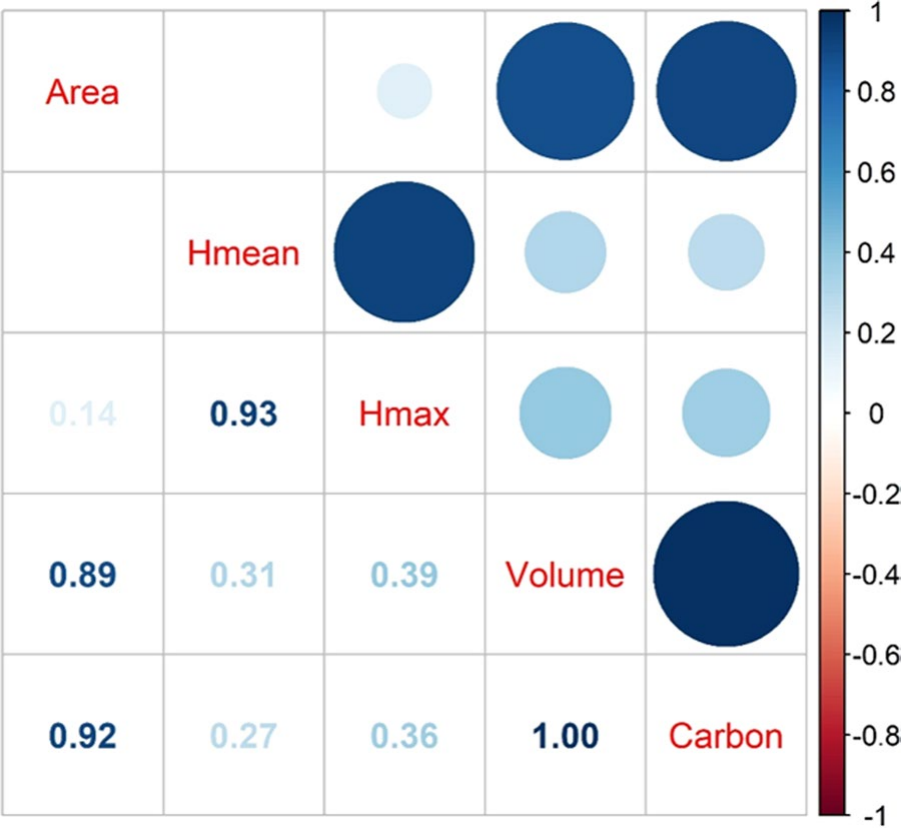
The correlation matrix among five tree structure variables over the six million trees in NYC. Area is crown size area. Hmean and Hmax are the mean and maximum height within each tree crown. Volume and Carbon are the tree crown volume and carbon storage in each tree.

Individual tree top height (Fig. 2) was tallest in State Island (average ave = 16.9 m) and smallest in Brooklyn (ave = 12.8 m). Interestingly, the crown area had the opposite pattern, with the largest crown area in Brooklyn (ave = 19.0 m^2^) and smallest in State Island (ave = 15.7 m^2^). The spatial distribution of individual tree volume was similar to tree height, with higher values in Bronx (ave = 180.9 m^3^) and State Island (ave = 178.0 m^3^). The differences in individual tree carbon storage among regions were smaller, with slightly higher values in Bronx (ave = 0.175 ton). A correlation among the five tree structural features (Fig. 3) at the individual tree level shows that there is a strong positive correlation between the mean and maximum tree height (r = 0.93). In addition, the tree crown area is highly correlated to tree crown volume (r = 0.89) and carbon storage (r = 0.92). The tree carbon storage is almost linearly correlated to tree crown volume (r = 0.99). To be noticed that these correlations were derived from all species combined, and these correlations could vary from species to species.

**Fig. 4:**
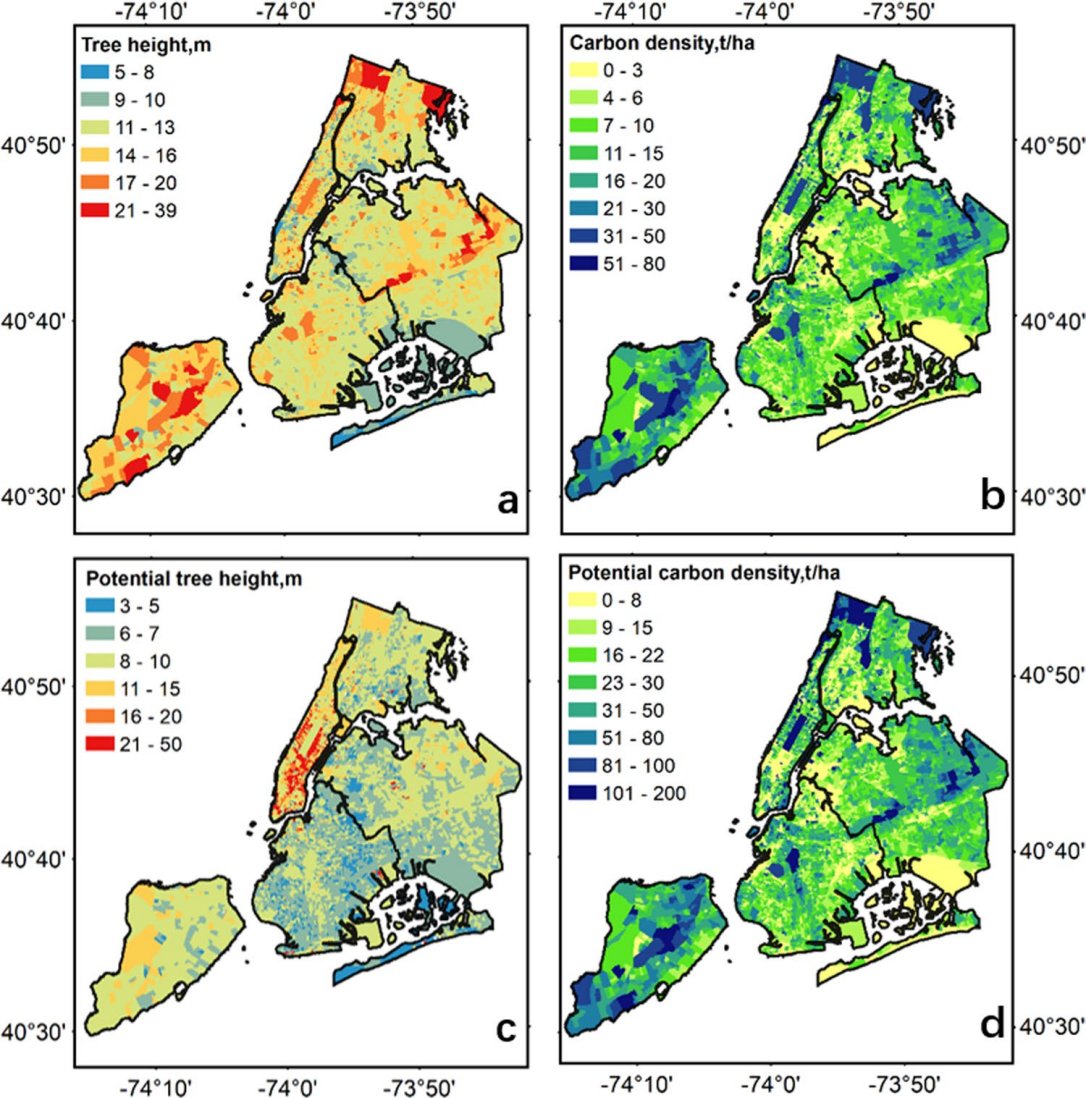
The map of mean tree height (**a**) and mean carbon density (**b**) over each census block group in NYC borough, and the potential increase in tree height (**c**) and carbon density(**d**) by calculating the difference between 95% and mean structural values within each block group.

Tree height (Fig. 4a) and carbon density (Fig. 4b) maps show similar spatial patterns at block group-level, where the tallest trees (>20 m) mainly located in the north Bronx, central Queens, and central and south State Island. These regions also contained the largest carbon density (>30 ton/ha). However, the potential for tree height growth (Fig. 4c) has different spatial patterns from the current tree height map (Fig. 4a). Some continuously large block groups in central-south of Manhattan have the largest potential for tree height increase (>15 m), whereas only a few small block groups that are randomly distributed in the other regions have a large potential for tree height increase. The potential for carbon density enhancement (Fig. 4d) from tree growth shows similar patterns with that from the current carbon density map (Fig. 4b), which indicates that places with dense trees and large carbon storage still have large potential for continued carbon storage.

## Technical Validation

The total number of trees (6,005,690) identified in this study was slightly smaller than the estimation from field sampling conducted by USFS, which was 6,977,000 trees with a standard sampling error of 874,000^9^. The difference in the total tree number can be attributed to two reasons: (1) estimation from USFS was based on field plot-measured trees with a DBH no less than 2.54 cm, whereas our dataset only counted trees with a top height larger than 5 m; (2) instead of using strategically located samples from 296 field plots, we identified the individual trees and their locations from wall-to-wall LiDAR-based CHM over the entire NCY directly. Considering the discrepancies in tree definition and estimation method, the two estimation results matched reasonably well.

In order to assess the commission and omission errors caused by the LiDAR CHM segmentation algorithm used in this study, we randomly selected 116 plots over this study area (Figure S2) and visually identified each tree crowns in the plots from LiDAR CHM. Similar to field plots measurement, each plot contains a circular area of 404.7 m^2^. In total, there were 485 tree crowns visually interpreted from LiDAR CHM and verified by three persons with expertise in remote sensing imagery interpretation. Among the 485 tree crowns, 378 were correctly segmented, 107 tree crowns were failed to be identified, and the omission rate was 22.1%. There were also 99 tree crowns falsely over-segmented, and the commission rate was 20.7%. Overall, this LiDAR CHM-based tree segmentation accuracy was 78.6% (F score) according to the accuracy assessment suggested in Li, *et al*.^27^.

Because individual tree locations in field surveyed plots are unavailable, we used plot-level total tree number, mean tree height, and maximum tree height to validate our LiDAR derived estimates. Starting from the field plot location, we shifted the center of plots within a 5 m range to consider the potential geolocation uncertainties in both field plot location and LiDAR data. The best match in the number of trees and averaged tree height was chosen for accuracy assessment. The tree number matched within each field plot is based on the number of trees with their central points located inside each plot polygon (a circle shape of 404.7m^2^) using the select by location function in ArcMap 10.8 software.

**Fig. 5:**
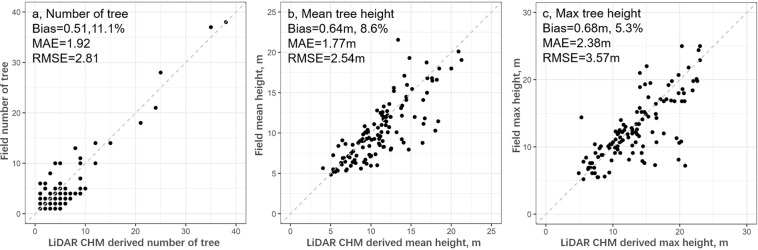
Validation of LiDAR derived number of tree in (**a**), mean tree height in (**b**), and maximum tree height in (**c**) using field plot measured values. The accuracies were evaluated based on bias (LiDAR estimate - Field measurement) and bias ratio (bias/Field measurement*100%), mean absolute error (MAE), and root mean squared error (RMSE), respectively.

We used the bias, bias rate, mean absolute error (MAE), and root mean square error (RMSE) to evaluate the accuracies related to LiDAR-derived tree height and tree number. Results showed that LiDAR-based results overestimated the number of trees by 0.51 on average, with a bias rate, MAE and RMSE being 11.1%, 1.92 and 2.81, respectively (Fig. 5). Comparing to the field-measured tree numbers, LiDAR CHM-derived tree segments over-predicted five trees in two plots and over-predicted four trees in nine plots. Majority of these plots contained many tall trees (mean height >10 m) with large tree crowns, where the marker-controlled region growth segmentation algorithm could over-segment single big tree crown into several smaller ones. These over-segmentations often occurred in deciduous tree species, such as maples and oaks that were dominant in NYC. Different from conifers that have an obvious tree top, the “fan-shape” tree crowns of deciduous could be over-segmented into several smaller tree crowns from LiDAR CHM. There were also six plots where LiDAR CHM under-segmented by 4–6 trees referencing to field measurements. These plots mainly contained short trees (mean height <6 m), and some of them were less than 5 m and were excluded in the LiDAR CHM-derived tree segments.

**Fig. 6:**
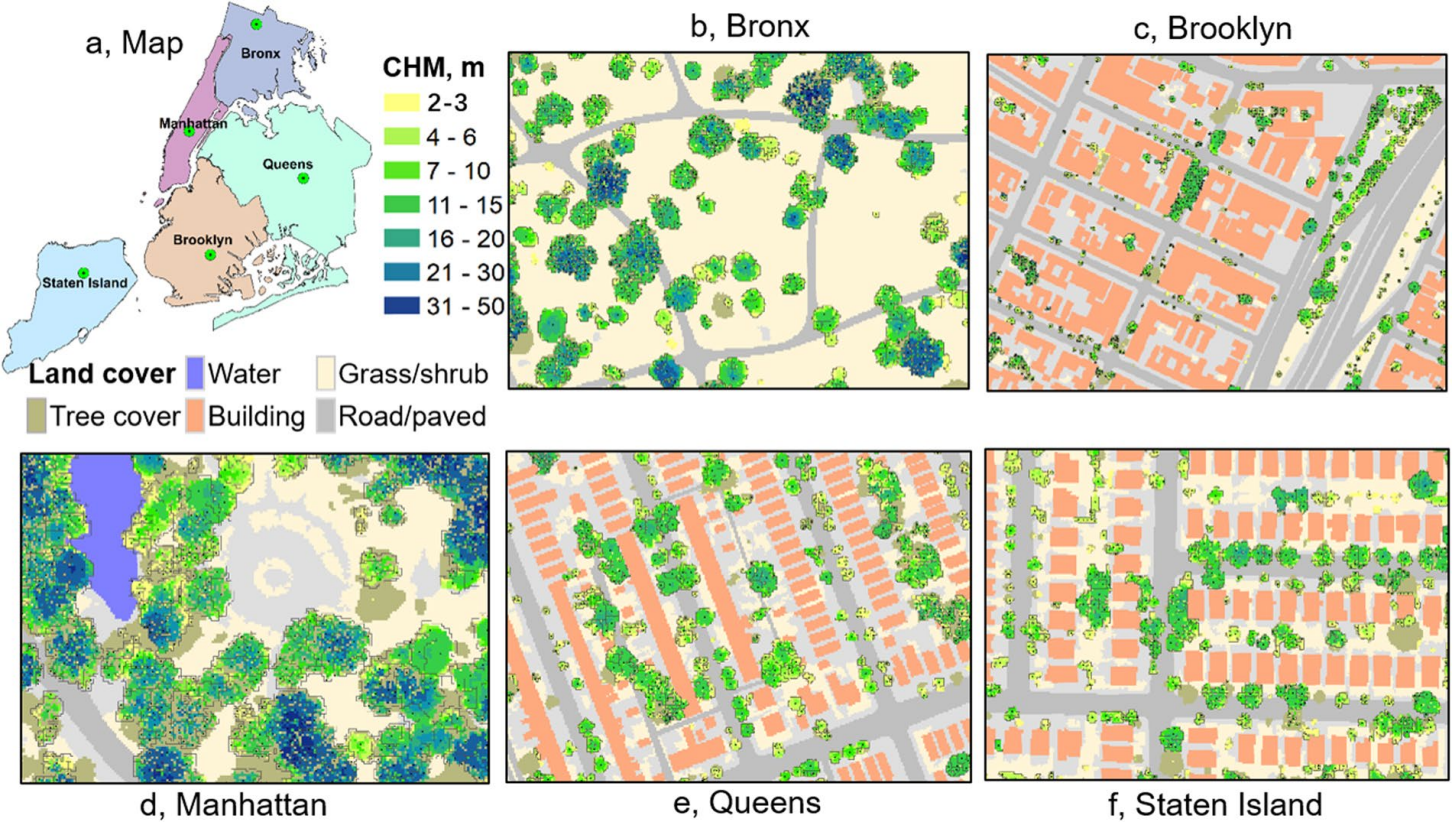
Examples of tree top locations (black dot) and crown segments overlaid on LiDAR-derived Canopy Height Model (CHM). The map in (**a**) shows the locations of example plot from five regions (**b**–**f**). Each plot represents an area of 180 m × 250 m, in typical city parks (**b**, Bronx and **d**, Manhattan) and industry (**c**, Brooklyn) and residential area (**e**, Queens and **f**, Staten Island). The background color is from the land cover map obtained in 2010. The CHM-derived tree segment was colored by their CHM values and isolated by tree crown boundaries.

LiDAR-derived tree height overestimated the mean and maximum tree height by 0.64 m and 0.68 m respectively, which accounted for an 8.6% and 5.3% bias ratio, respectively. The MAE and RMSE were 1.77 m and 2.54 m for mean tree height, and 2.38 m and 3.57 m for maximum tree height, respectively. The field-measured plot mean tree height was largely under-predicted by LiDAR CHM only in one plot (bias = −8.2 m), while the opposite was observed at six plots (bias >5 m). Similarly, the field-measured plot maximum tree height was largely under-predicted by LiDAR CHM in five plots (bias <−5 m), and largely over-predicted in eleven plots (bias >5 m). These under-predications mainly occurred in plots that contained a small number of tall trees where a slight mis-match in plot locations can cause the failure of locating the same tall trees from LiDAR CHM. In contrast, the over-predictions can be caused by two main reasons: 1) The tallest part of tree crown could be missed in the field measurements, particularly for deciduous trees with dense canopy cover. LiDAR data were collected from aircraft above the canopy, and thus were less likely to miss the tree tops. 2) The land cover map we used to mask out non tree cover was generated in 2010, which is three years before the LiDAR data and field measurements were collected (2013). Some new built-ups features, such as building roofs and utility poles, could be incorrectly identified as tree tops in the LiDAR CHM, and over-predicted tree height (Fig. 6). Overall, the geolocation errors from remotely sensed datasets and field measurements, as well as differences in data collection methods could make the precise identification of trees difficult, and thus lead to discrepancies in field measurements and our estimations of tree number and heights (Fig. 6).

The tree crown volume could be sensitive to the selection of tree crown base height, which varied by individual tree species and age. We assessed the uncertainty of tree crown volume caused by different crown base heights from 1 m to 5 m with a 1 m interval. The 1 m to 5 m was considered as reasonable range of tree crown base height in NYC and the corresponding tree crown volume estimates are presented in Table 2 and Figure S1. The mean crown volume for the 2 m cut-off used in the final product was 175.4 m^3^, but it varied from 192.4 m^3^ to 124.6 m^3^ using the 1 m to 5 m cut-offs for crown base height, respectively. These values can be used as a reference of uncertainty ranges for tree crown volume estimation caused by crown base height selection in NYC.

**Table 2:** A summary of tree crown volume over the NYC using the crown base height (CBH) value from 1 m to 5 m. The first quartile (q25), median, mean, and third quartile (q75) values of tree crown volume are presented.

*Tree crown volume, m*^3^					
	*CBH1m*	*CBH2m*	*CBH3m*	*CBH4m*	*CBH5m*
q25	38.2	33.3	27.9	22.0	16.0
Median	93.9	83.3	72.6	64.0	51.7
Mean	192.4	175.4	158.4	141.4	124.6
q75	234.2	212.3	190.7	169.3	148.4

**Fig. 7:**
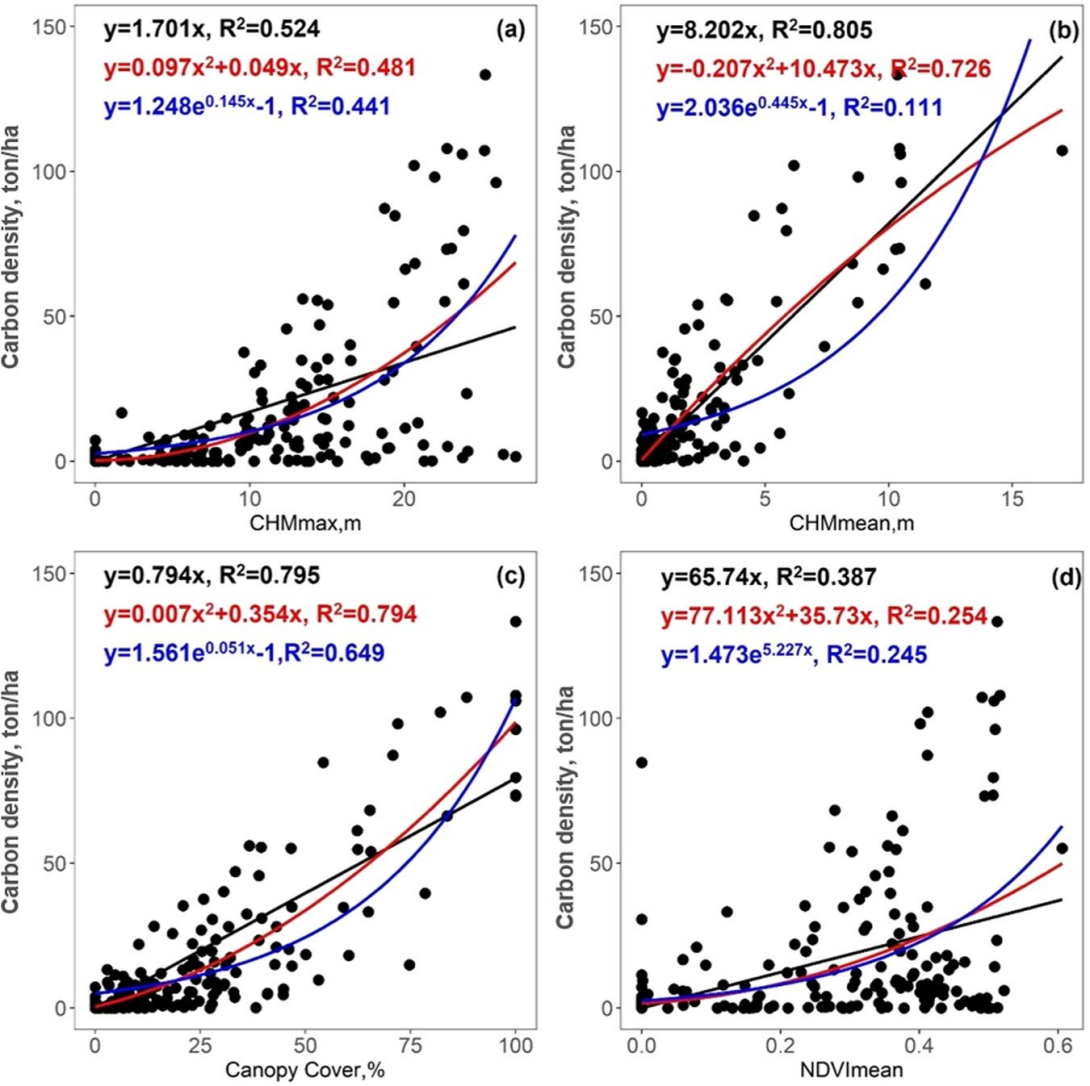
Carbon density regression functions fitted from LiDAR-derived CHMmax (**a**), CHMmean (**b**), Canopy Cover (**c**), and NAIP imagery derived NDVImean (**d**), using linear (black), quadratic (red), and exponential (blue) functions, respectively.

The carbon density model was determined from the relationships between plot-measured carbon density and metrics derived from LiDAR data and NAIP imagery (Fig. 7). The mean CHM value over plots (CHMmean) showed the strongest linear correlation with carbon density (coefficient of determination R^2^ = 0.81), better than non-linear regression using either quadrate (R^2^ = 0.73) or exponential (R^2^ = 0.11) regression functions. The LiDAR-derived canopy cover also has strong correlations with carbon density (R^2^ = 0.80), but the regression was limited by a saturation effect. As the canopy cover would not be larger than 100%, the canopy cover-based carbon density estimation would be less than 79.4 ton/ha, which was unreasonable according to field measurements. The regression models from other metrics, such as CHMmax and NDVImean or their combination were not better than the linear regression model from CHMmean (R^2^ ≤ 0.76). We used the 10-folded cross-validation method to test the model estimation accuracy by using 90% of field plots as training, and the rest 10% as validation and repeated 10 times. The results confirmed that the CHMmean linear model had the best performance (averaged R^2^ = 0.73), followed by Canopy Cover linear model (averaged R^2^ = 0.71), and CHMmax (averaged R^2^ = 0.43). The CHMmean based linear model also gives the lowest RMSE at 10.82 ton/ha, closely followed by the Canopy Cover model (RMSE = 10.91 ton/ha). Therefore, we adopted the CHMmean linear model for carbon density estimation at plot level. More details about the carbon density model comparison and validation can be found in our previous study^42^.

**Fig. 8:**
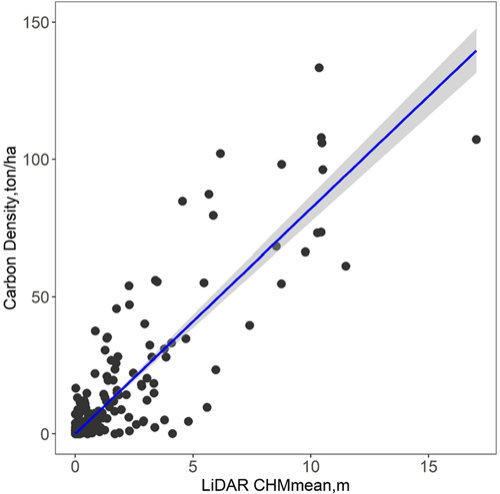
Carbon density prediction model based on the mean LiDAR CHM developed from field sampled plots (black dots). The linear regression was selected as the optimal model (blue line) along with the 95% confidence interval (grey shadow).

The uncertainty in the final carbon density regression model is presented in Fig. 8 with the grey shadow indicating the 95% confidence interval. We further used this linear relationship between carbon density and LiDAR CHMmean to calculate individual tree storage along with their uncertainty range. The uncertainty range of individual tree carbon storage that propagated from carbon density regression is summarized in Table 1. The averaged carbon storage of all trees in NYC was 0.172 ton, with a lower and upper values of 0.162 and 0.182 ton, respectively. The uncertainty range was larger in trees with high carbon storage. Several sources could have contributed to the uncertainties, such as the spatial mis-match between field plots and LiDAR dataset, the lack of individual tree species information in biomass estimation, and bias in carbon density regression fitting.

## Usage Notes

Researchers can use our Trees in New York City datasets to calculate vegetation density and biomass and assess their ecosystem values at different spatial scales, analysing urban tree’s cooling effects at three-dimensional scales, and making practical tree protection and management plans.

As this suit of dataset is spatially explicit, we also recommend overlaying these maps with other geo-referenced datasets, such as building-footprints, environmental pollutants, census datasets, and climate features for further analysis and applications at multiple scales. These analyses can be achieved via GIS software and programming packages, such as ArcGIS, SAGAGIS, QGIS, GDAL, and GeoPandas.

## Supplementary information

Supplementary document

## Data Availability

We used SAGA GIS, ArcMap, LiDAR360 software to process all the remotely sensed datasets. We performed all other steps including biomass modelling, data analysis, and figure generations in R. All the codes along with input datasets can be found in in the following link: https://github.com/qinmaNNU/NYCtrees. This github folder includes codes developed for: 1. carbon storage prediction and uncertainty estimation (CarbonDensityUncertainty_Fig. 7Fig. 8Table 1.R); 2. tree structural distribution analysis over regions and their correlations among structural features (NYtree_Fig. 2RegionViolin_Fig. 3CorrMatrix.R); and 3. validation of tree structural estimation using field plot measurements (NYtreesVali_Fig. 5.R).
