# Supplementary material for: Individual structure mapping over six million trees for New York City USA
Source: Sci Data. 2023 Feb 20;10:102. doi: 10.1038/s41597-023-02000-w (PMC9941473; doi:10.1038/s41597-023-02000-w)
Supplement: Supplementary file 1 — Supplementary document [file 41597_2023_2000_MOESM1_ESM.docx]

Supplementary for “Individual structure mapping over six million trees for New York City, USA”


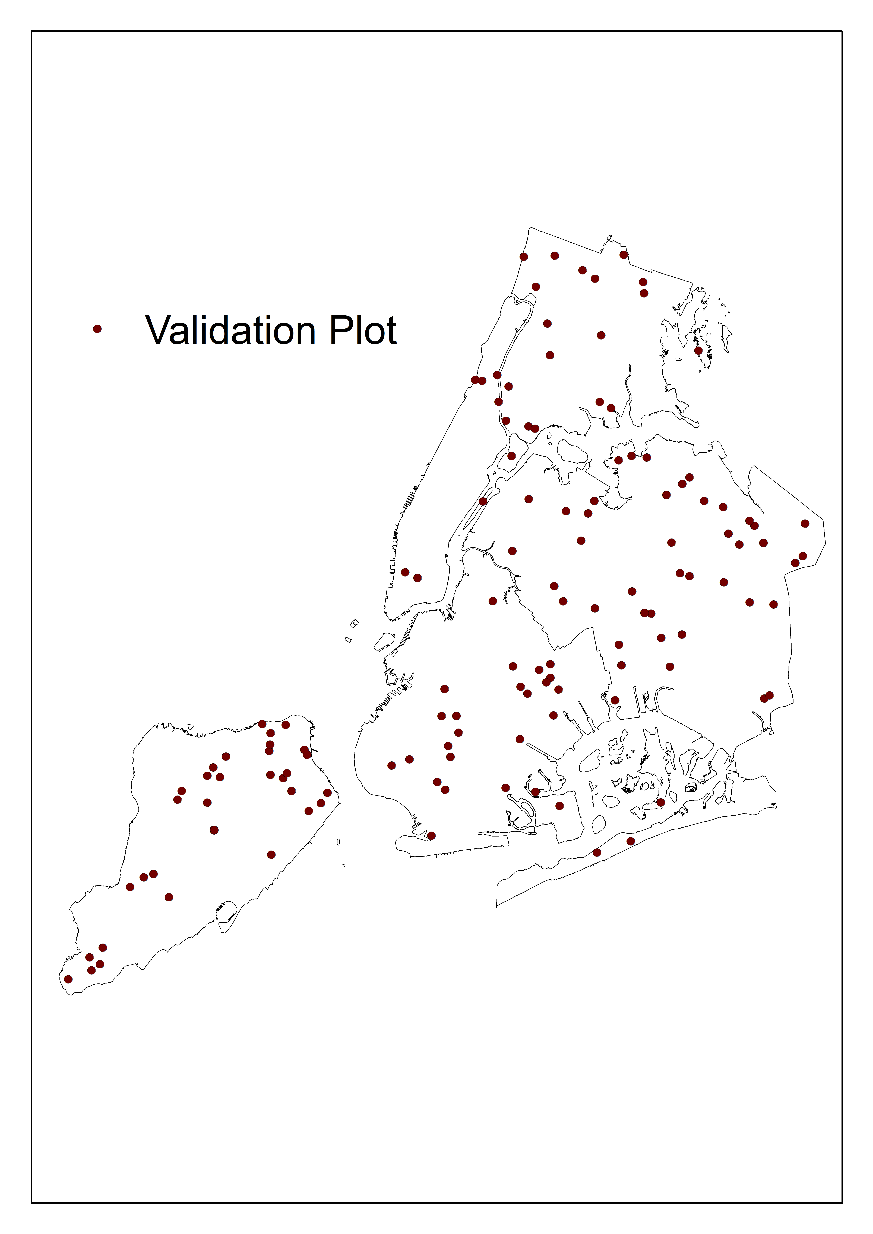


Figure S1 map of 116 randomly distrubted plots over New York City for the accuracy assessment of tree segmentation from LiDAR derived Canopy Height Model (CHM).


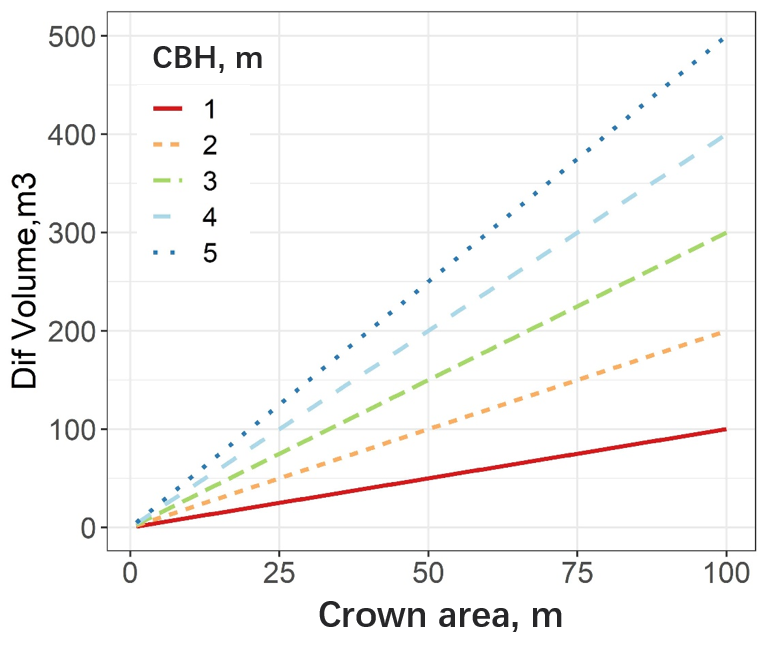


Figure S2 The changes in volume under crown base height (Dif Volume = Volume under LiDAR Canopy Height Model– Crown volume) caused by the crown base height (CBH) selection and crown area.
